# A Rare Partner of TFE3 in the Xp11 Translocation Renal Cell Carcinoma: Clinicopathological Analyses and Detection of MED15-TFE3 Fusion

**DOI:** 10.1155/2019/5974089

**Published:** 2019-11-11

**Authors:** Hong Ye, Shuming Qin, Ning Li, Min Lin, Yanmei Xu, Xiaomei Li

**Affiliations:** ^1^Department of Pathology, Tai'an Central Hospital, Tai'an 271000, China; ^2^Department of Pathology, Shanxian Central Hospital, Heze 274300, China

## Abstract

Xp11 translocation renal cell carcinoma (RCC), a member of the microphthalmia-associated transcription factor (MiTF) family, is a rare renal tumor characterized by different translocations involving the *TFE3* gene. Here, we reported a case of Xp11 translocation RCC with a rare *MED15-TFE3* gene fusion by RNA sequencing. Morphologically, the tumor cells were arranged in a solid and small nest pattern. The cytoplasm was voluminous, flocculent eosinophilic, and vacuolated. The nuclei were round or polygon with fine granular chromatin, and the nucleoli were unconspicuous. Psammoma bodies were observed in mesenchyma. Immunohistochemically, the tumor cells were diffuse moderately or strongly positive for CD10, P504S, vimentin, PAX8, RCC, AE1/AE3, and SDHB and focally positive for CK7 and CA IX while negative for cathepsin K, HMB45, Melan-A, Ksp-cadherin, and CD117. The Ki67 proliferation index was approximately 3%. However, TFE3 labeling showed an uncertainly weak nuclear staining and been considered negative. Fluorescence in situ hybridization (FISH) demonstrated a positive result that splits signals with a distance of > 2 signal diameters. Subsequently, RNA sequencing confirmed a fusion of *MED15* gene exon 11 on chromosome 22 with *TFE3* gene exon 6 in the tumor. The patient was alive with no evidence of recurrence. Our report contributes to the understanding on *MED15-TFE3* RCC.

## 1. Introduction

Microphthalmia-associated transcription (MiT) family translocation renal cell carcinoma (tRCC), consisted of Xp11 tRCC and t(6; 11) RCC, is a distinct cancer subtype named in 2016 WHO classification of renal tumor [1]. Xp11 tRCC is featured by a short arm of X chromosome translocation with other chromosomes, leading to *TFE3* gene fusion. It is commonly reported in children and young adults, especially in young women, accounting for about 40% of RCC in children and 1.6–4% in adults [2–4]. The morphological manifestations of Xp11 tRCC are diverse. Additionally, they are occasionally confused with other common types of RCCs. *TFE3* translocation-associated RCC was divided into different genotypes according to the target genes of the translocation. The morphological, immunophenotype, and prognosis of different subtypes were different and distinct. However, inadequate description has been given on it by WHO histological classification of RCC due to rarity. Therefore, its pathological diagnosis is still a challenge. To make a definitive diagnosis, several techniques are required including detection of *TFE3* antibody based on the immunohistochemical method, fluorescence in situ hybridization (FISH) detection of *TFE3* fusion gene, and RNA sequencing. The relatively common fusion couples of *TFE3* gene included *ASPSCR1*, *PRCC*, *SFPQ*, and *NONO* [5–9]. On rare conditions, *TFE3* may fuse with other couples including *CLCT*, *RBM10*, *PAPR14*, *MATR3*, *LUC7L3*, *FUBP1*, *DVL2*, *KHSRP*, *GRIPAP1*, and *MED15* [10–19].

In this study, we reported a case of *MED15-TFE3* RCC confirmed by high-throughput RNA sequencing. Also, FISH involving the utilization of *TFE3* break-apart probes was performed. Our data confirmed that the histological morphology and immunophenotype were different from the features described in the previous literatures [17, 18].

## 2. Materials and Methods

### 2.1. Immunohistochemistry Staining

Tissue samples were obtained from a female patient with left-sided renal cancer. For the immunohistochemical analysis, tissues were fixed on 10% formalin, followed by embedding using paraffin. Then, the tissue sections (4 *μ*m) were treated with the Ventana BenchMark XT automated IHC stainer (Roche, Basel, Switzerland). Sections treated with PBS served as the negative control. As a positive control for TFE3, we selected one renal carcinoma associated with the Xp11.2 translocation presenting the *TFE3* gene translocation by FISH. While the positive control of others was using the specific tissues according to the manufacturer's instructions. The protein antibodies utilized in this analysis were as follows: TFE3 (ab179804, 1 : 300; Abcam), cathepsin K (3F9, 1 : 300; Abcam), SDHB (21A11AE7, 1 : 300; Abcam), PAX8 (polyclone, 1 : 800; Proteintech Group, Chicago, Illinois, USA), HMB45 (polyclone, 1 : 50; Dako), Melan-A (A103, 1 : 100; Novocastra Laboratories, Newcastle, UK), CD10 (56C6, prediluted; Novocastra Laboratories, Newcastle, UK), vimentin (V9, 1 : 200; Dako), CK7 (OV-TL 12/30, 1 : 50; Dako), Ksp-cadherin (ab80320, 1 : 800; Abcam), P504S (13H4, 1 : 200; Dako), RCC (PN-15, prediluted; Ventana Medical System Inc, Tucson, AZ, USA), CKpan (AE1/AE3, 1 : 100; Dako), CD117 (2E4, 1 : 1000; Dako), and Ki-67 (MIB-1, 1 : 50; Dako).

TFE3 nuclear immunoreactivity was scored from 0 to 3 + referring to the criteria proposed by Argani [20]. The criteria were as follows: 2 + and 3 + were positive; 1 + was considered negative.

Immunohistochemical evaluation of other antibodies was based on the staining percentage of positive cells: 0–5%, negativity; 6–10%, focal positivity (+); 11–50%, moderate positivity (++); >50%, diffuse positivity (+++).

### 2.2. FISH

Hematoxylin and eosin-stained tissue slides were prepared and were observed under a microscope to count the tumor cells in the selected fields. Upon deparaffinization with xylene thrice, the slides were washed twice with absolute ethanol. Digestive enzyme K was incubated with 40 ml 2 × SCC at 37°C for 20 min. The mixture was rinsed thrice at room temperature and was dehydrated with precooling gradient alcohol. *TFE3* break-apart probe (EmpireGenomics, Buffalo, NY) was used. The centromere side was labeled with green fluorescence, while the telomere side was labeled with red fluorescence. The 10 *μ*l probe was dropped on the slide and then was quickly covered with a 2,222 mm cover glass. Afterwards, the section was slightly pressed for the even distribution of the liquid, followed by sealing with rubber cement. Denaturation was conducted by incubating the slides at 85°C for 5 min in a humidified box followed by hybridization at 37°C overnight. After removal of cover glass, the slide was washed with 0.1SSC/1.5M urea at 37°C for 10 min. Subsequently, the slide was washed again using 2 × SSC/0.1% NP-40 for 5 min at 37°C. The slides were put into 70% ethanol thrice and were air dried. The nuclei were counterstained with 4, 6-diamidino-2-phenylindole. After hybridization, all slides were maintained at 4°C in the dark.

At least 100 tumor nuclei with clear boundaries and no overlap were counted by fluorescence microscopy under a magnification of 1,000×. Positivity was defined as a distance of separation between red and green signals of more than 2 signal diameters in more than 10% tumor cell nuclei.

### 2.3. High-Throughput RNA Sequencing

RNA was extracted from the paraffin-embedded cancer tissues, and the RNA concentration quantified with Qubit precisely; RNA purity detected by Nanodrop, Agarose gel electrophoresis and Agilent 2100 Bioanalyzer was used to detect RNA integrity. Magnetic beads with oligo-dT were utilized to purify the mRNA. Agencourt SPRIselect Reagent kit was used for the purification of libraries and selection of fragments. Library concentration and library fragment length distribution were accessed using the Qubit and Agilent 2100 Bioanalyzer, respectively. High-throughput sequencing was performed on Illumina Hiseq platform with 2 × 150 bp double-end sequencing mode to get FastQ data. FusionCatcher and BLAT aligner algorithms were applied for the detection of any potential *TFE3* fusion of RNA-sequencing data.

## 3. Results

### 3.1. Clinical Information

**Figure 1:**
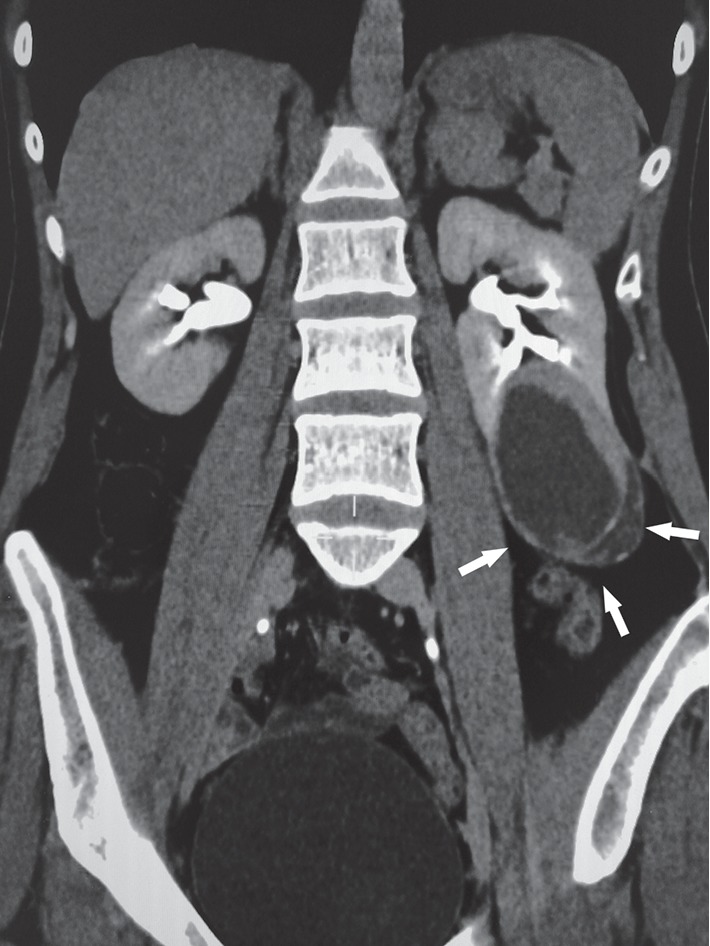
CT scan revealed that there was a cystic solid lesion at the lower pole of her left kidney, which protruded the renal parenchyma with a clear boundary (arrow).

The patient was a 35-year-old woman presented to our department due to fatigue without other symptoms. She showed a history of hepatitis B for more than 10 years. There was no family history of tumor. Abdominal computed tomography (CT) scan revealed a cystic solid density lesion at the lower pole of her left kidney, which protruded the renal parenchyma with a clear boundary. Imaging diagnosis confirmed RCC with a Bosniak grade of 3–4 (Figure 1). Radical nephrectomy was performed. Macroscopy showed a well-circumscribed cystic solid mass at the lower pole of the kidney with a tumor size of 5 × 4 cm in a grayish red color of a tough texture. For the TNM staging, the patient was in a stage of T1N0M0, clinical stage I. The patient was still alive without occurrence after nephrectomy.

### 3.2. Histopathology

**Figure 2:**
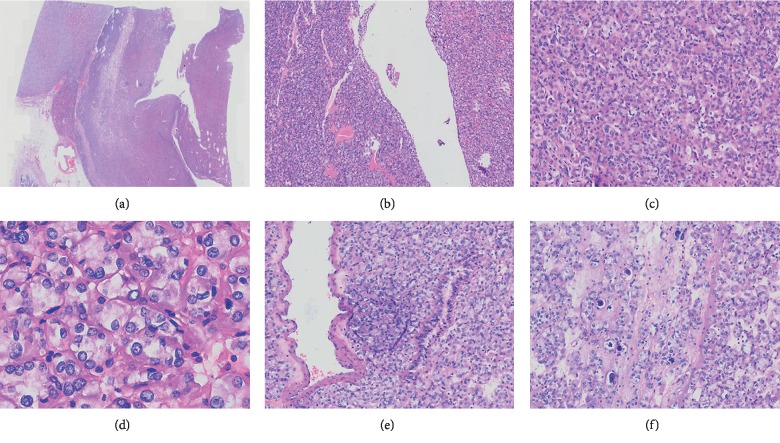
Pathological findings. (a) The tumor was well defined with a pseudocapsule invasion into the capsule locally, and cystic structures were visible in some areas (0.25×). (b) The capsule wall was lined with flattened tumor cells or fibrocystic wall (10×). (c) The tumor cells were arranged in a profile of solid and small nests (10×). (d) The cytoplasm was voluminous, flocculent eosinophilic, and vacuolated (40×). (e) Normal renal tubules were involved in the tumor focally (10×). (f) Psammoma bodies were observed in mesenchyme (10×).

Microscopically, the tumor was well defined with a pseudocapsule invasion into the capsule locally at a low magnification of 0.25×. Most parts of the tumor were solid and cystic structures were visible in some areas (Figure 2(a)). Under a higher magnification of 10×, the capsule wall was lined with flattened tumor cells or fibrocystic wall (Figure 2(b)). The tumor cells were arranged in a profile of solid and small nests (Figure 2(c)). The cytoplasm was voluminous, flocculent eosinophilic, and vacuolated (Figure 2(d)). The nuclei were round or polygon with fine granular chromatins, and the nucleoli were unconspicuous. Normal renal tubules were involved in the tumor focally (Figure 2(e)). In the mesenchyma of cancer tissues, there was a lack of capillary network, and psammoma bodies were observed (Figure 2(f)).

### 3.3. Immunohistochemistry

**Figure 3:**
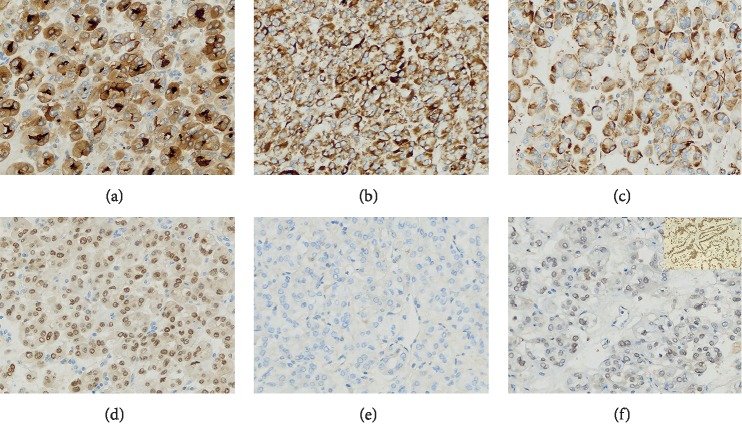
Immunohistochemistry findings of TFE3. (a–d) The tumor cells were diffusely and strongly positive (+++) for CD10, SDHB, vimentin, and PAX8. (e) The tumor cells were negative for cathepsin K. (f) TFE3 showed an uncertainly weak nuclear staining (1+); the upper right corner was the positive control.

Immunohistochemically, the tumor cells were diffuse and strongly positive (+++) or moderately positive (++) for CD10, P504S, vimentin, PAX8, RCC, AE1/AE3, and SDHB. Focally positive (+) was observed for CK7 and CA IX. Staining results for cathepsin K, HMB45, Melan-A, Ksp-cadherin, and CD117 were negative. The Ki67 index was approximately 3%. Particularly, TFE3 showed a weak nuclear staining (1+) and was considered negative (Figures 3(a)–3(f)).

### 3.4. FISH

**Figure 4:**
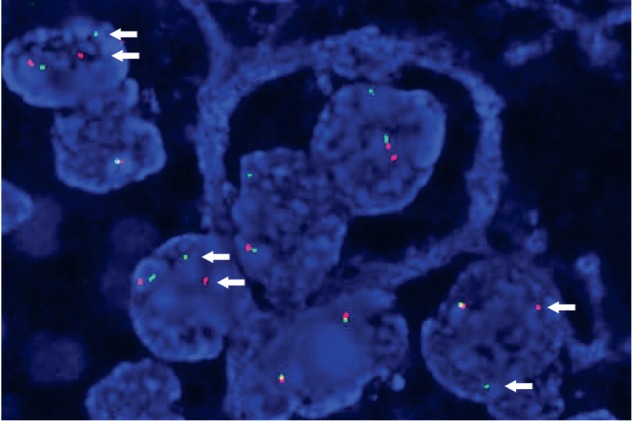
FISH results. TFE3 break-apart assay displayed green and red signals were split (indicated by an arrow).

Because of the indeterminacy of TFE3 immunohistochemistry stain, dual-color FISH using a *TFE3* break-apart probe was examined. The result indicated that the distance of green and red signals surpassed the width of more than 2 signals. In total, 129 cells were counted and signals split were detected in 65 cells (50.39%), indicating the fusion of *TFE3* gene (Figure 4).

### 3.5. RNA Sequencing

**Figure 5:**
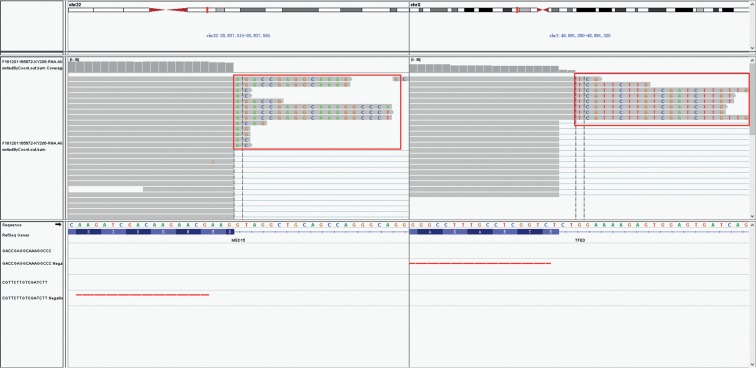
RNA-sequencing results. RNA sequencing showed a fusion of *MED15* gene exon 11 on chromosome 22 with *TFE3* gene exon 6 on chromosome X.

To further investigate the gene partner fused with *TFE3*, high-throughput RNA sequencing was performed. Using both the FusionCatcher and BLAT aligner algorithms, we could detect 10 reads on *MED15* and 5 reads on *TFE3*, suggesting a fusion of *MED15* gene exon 11 on chromosome 22 with exon 6 of *TFE3* gene on chromosome X. Fusion abundance was 38% (Figure 5).

## 4. Discussion

Renal carcinoma associated with Xp11.2 translocations/*TFE3* gene fusions is an unusual renal tumor, which is recognized as an entity in 2004 WHO classification of tumors of the urinary system [1, 21]. The majority (40%) of paediatric RCCs are Xp11 translocation RCCs, whereas approximately 1.6–4% of adult RCCs are Xp11 translocation RCCs [2–4]. The most common histological pattern of the Xp11 translocation RCCs is that of a papillary neoplasm composed of epithelioid clear cells with abundant psammoma bodies [5]. Xp11 translocation RCCs can also resemble other renal neoplasms, including clear cell RCC, papillary RCC, multilocular cystic renal neoplasm of low malignant potential, oncocytoma, and epithelioid angiomyolipoma [17, 18, 22]. Xp11 translocation RCCs harbors fusions of the *TFE3* transcription factor gene with one of multiple reported genes including *ASPSCR1*, *PRCC*, *NONO/p54nrb*, *SFPQ/PSF*, and *CLTC* [5–8, 14]. With the development of research, more and more gene combinations were discovered in Xp11 translocation RCCs. In this study, we report a rare case of *MED15-TFE3* RCC. There were only 7 cases of *MED15-TFE3* RCCs in the previous literatures [17, 18, 23]. Classe et al. [17] reported the first case of *MED15-TFE3* RCC confirmed by RNA sequencing in 2017. Wang et al. [18] represented 5 cases of *MED15-TFE3* RCC by RNA sequencing and fusion FISH probe. Recently, Pei et al. [23] also found a case of *MED15-TFE3* RCC. The clinical characteristics and immunohistochemical profiles of the cases in literature, along with the case in this study, are summarized in Table 1. Clinically, the tumors tended to occur in young patients (ranged from 22 to 54 yrs; mean: 37 yrs) and showed female predominance. The mean size was 5.5 cm (ranged 1.5–9.5 cm). In morphology, Classe et al. [17] described the tumor as a mixed structure of papillary, solid, and cystic. In the 5 cases reported by Wang et al. [18], three cases were extremely similar to multilocular cystic renal neoplasm of low malignant potential. One case demonstrated a mixture of cystic areas, papillary, and solid structures. In another case, tumor cells were arranged in acinar, tubular, and papillary patterns. All tumors were composed of cells with clear or granular cytoplasm. Pei et al. [23] showed the tumor presented papillae and granular cytoplasm with clearing; however, no psammoma bodies were identified. Immunohistochemically, all cases in documents were strongly positive for TFE3, six cases were positive for PAX8, as well as diffusely positive for melanocytic markers cathepsin K and Melan-A. In this study, we presented a case with cystic and solid construction under macroscopic condition and the capsule wall was lined with flattened tumor cells or fibrocystic wall under the microscope. The tumor was composed of clear or eosinophilic cells arranged in a solid and small acinar to nest pattern, which showed a relative simple structure compared with the mixed structures of papillary, acinar, and cystic. The cytoplasm of the tumor cells was voluminous, flocculent eosinophilic, and vacuolated mimicking SDHB-deficient RCC and with small nuclei without nucleoli (WHO/ISUP grade 1). Psammoma bodies were observed in mesenchyme. Interestingly, there were normal renal tubules involved in tumors, which had not been mentioned previously and showed some differences in histomophology. In addition, the immunotype of the case was also different from that reported previously [18, 23], as the neoplastic cells were negative for cathepsin K, HMB45, Melan-A, and TFE3 which showed an uncertainly weak nuclear staining and considered negative.

Cathepsin K is a cysteine protease from the papain family playing an important role in osteoclast function. Expression of cathepsin K in osteoclasts is regulated by MITF [24]. Cathepsin K was demonstrated to be a transcriptional target of the microphthalmia-associated transcription factor family, which revealed that Cathepsin K was differentially expressed depending on the fusion partner of the *TFE3* gene [25, 26]. In the known genotypes, *PRCC-TFE3* gene fusion showed a positive reaction with antibody to cathepsin K, while *ASPL-TFE3*, *SFPQ-TFE3*, and *NONO-TFE3* gene fusion showed negative staining [25]. Martignoni et al. [26] speculated that different expression of Cathepsin K between subtypes of Xp11 translocation renal cell carcinomas might be contributed to some of clinical, pathological, and biological differences. Cathepsin K expression was strongly and diffusely positive in all the 6 rare *MED15-TFE3* gene fusion cases previously reported [18]; however, it was negative in our case. We suspected this difference in expression was related to the simplicity of the morphological structure.

TFE3 antibody is a characteristic marker of Xp11 translocation RCCs for the overexpression of the functional TFE3 fusion protein. However, immunohistochemical methods are susceptible to false-positive, false-negative, and uncertain results due to various factors, such as tissue fixation time, antigen retrieval mode, antibody clonal number, and data interpretation [27]. In this study, TFE3 showed a weak nuclear staining and was considered negative, while the positive control was strong positive, indicating the reliability of staining results and excluded false negatives caused by antibody clonal number. Rao et al. [27] compared the sensitivity and specificity of TFE3 immunohistochemical staining and *TFE3* break-apart FISH assay in the diagnosis of Xp11 translocation RCCs. Their data showed that *TFE3* break-apart FISH assay was a useful complementary method for confirming the diagnosis of Xp11 translocation RCC, especially when the morphologic or clinical suspicion was high but TFE3 immunostaining was negative or equivocal. TFE3 immunohistochemical staining was negative in our case; thereupon, *TFE3* break-apart FISH assay was performed and the presence of *TFE3* translocation was confirmed.

To further investigate the gene partner fused with *TFE3*, we performed high-throughput RNA sequencing. Our data confirmed that *MED15-TFE3* RCC was an extremely rare fusion genotype. RNA sequencing showed *MED15* exon 11 fused with *TFE3* exon 6. *MED15* was a part of the multiprotein mediator complex, which functioned as a bridge between regulatory proteins and RNA polymerase II (Pol II), thereby regulating the Pol II-dependent transcription [28, 29]. According to the previous studies, MED15 was overexpressed in 35% of primary head and neck squamous cell carcinoma and was known to be involved in castration-resistant prostate cancer [30–32]. *MED15-TFE3* fusion was also obtained in one case of melanotic Xp11 neoplasm [18]. Recently, it was reported to be a promoter of tumor progression and metastatic spread in renal cell carcinoma [33]. In future, further studies are required to investigate the role of *MED15* in Xp11 translocation RCCs.

The clinical manifestations of Xp11 translocation RCCs included hematuria, abdominal mass, abdominal pain, and weight loss. These conditions showed no obvious specificity compared with other renal tumors. The survival time for patients with Xp11 translocation RCCs was similar to that with clear cell RCCs [34]. Compared with the patients with papillary RCCs, their survival time was significantly shorter [35]. Caliò et al. [36] reviewed 403 cases of Xp11 translocation RCCs described in the literatures and considered that there was no statistical difference of age between aggressive and nonaggressive cases, while a larger tumor size correlated with aggressive behavior [36]. More patients with *ASPSCR1-TFE3* RCCs tended to show metastasis than those with *PRCC-TFE3* RCCs (75% vs. 36%) [37]. However, most of the node-positive *ASPSCR1-TFE3* RCC patients remained disease-free without adjuvant therapy [2, 34]. Hence, locally advanced stage may not predict adverse outcomes. To our best knowledge, only 8 cases of *MED15-TFE3* RCC were reported including our case. Five cases were symptom free in the 2–48 months follow-up, one case showed lung metastases after 15 years, and one case had no follow-up data [17, 18, 23]. The present case showed a low nuclear level in histology and followed up for 5 months with no evidence of recurrent disease. As the sample size was indeed small and the follow-up duration was short, long-term follow-up was still required to investigate the prognosis and true biological behaviors. Radical resection is one of the optional methods similar to that for conventional RCC. Adjuvant therapy is feasible, such as immunotherapy using cytokines, including interleukin-2 and interferon-alfa, but the curative effect is different [3]. In recent years, *TFE3/IRS-1/PI3K/AKT/mTOR*, as a potential dysregulated pathway in *TFE3*-tRCC, may serve as a therapeutic potential for vertical inhibition of such axis by using a dual *PI3K/mTOR* inhibitor for *TFE3*-tRCC patients [38].

There are indeed some limitations in this study. Firstly, the sample size is not large due to disease rarity. Secondly, we cannot bring new information to the molecular mechanism of the disease. Our study is merely a report of a unique disease with a different subset.

## 5. Conclusions

In this article, we described *MED15-TFE3* RCC, a rare gene subtype of Xp11 translocation RCCs, that was confirmed by FISH and RNA sequencing. The tumor demonstrated different morphological features and immunophenotypic characteristics with the cases reported in literatures, expanding our understanding on heterogeneity of *MED15-TFE3* RCC. FISH analysis is an accurate and effective approach for the screening and confirmation of this tumor in the presence of uncertain TFE3 expression. RNA sequencing will help to identify this specific gene fusion subtypes, leading to more accurate diagnosis and better understanding of such type of tumor.

## Figures and Tables

**Table 1:** Clinical characteristics and immunohistochemical profiles of 8 cases of *MED15-TFE3* RCC.

Case no.	Age	Gender	Size (cm)	Immunohistory					*TFE3* FISH	RNA-seq
				TFE3	PAX8	Cathepsin K	HMB45	MelanA		
1	34	F	9.5	+	NA	NA	NA	NA	+	+
2	42	M	1.5	+	+	+	+	+	+	+
3	41	M	9	+	+	+	+	+	+	+
4	54	F	4	+	+	+	+	+	+	+
5	45	F	4.5	+	+	+	+	+	+	NA
6	30	F	5	+	+	+	+	+	+	NA
7	22	F	NA	+	+	+	−	+	NA	+
Current case	35	F	5	−	+	−	−	−	+	+

NA, not available.

## Data Availability

All the data were available upon appropriate request.

## References

[1] Moch H., Humphrey P. A., Ulbright T. M., Reuter V. E. (2016). *WHO Classification of Tumours of the Urinary System and Male Genital Organs*.

[2] Sukov W. R., Hodge J. C., Lohse C. M. (2012). TFE3 rearrangements in adult renal cell carcinoma: clinical and pathologic features with outcome in a large series of consecutively treated patients. *The American Journal of Surgical Pathology*.

[3] Komai Y., Fujiwara M., Fujii Y. (2009). Adult Xp11 translocation renal cell carcinoma diagnosed by cytogenetics and immunohistochemistry. *Clinical Cancer Research*.

[4] Zhong M., De Angelo P., Osborne L. (2012). Translocation renal cell carcinomas in adults. *The American Journal of Surgical Pathology*.

[5] Argani P., Antonescu C. R., Illei P. B. (2001). Primary renal neoplasms with the ASPL-TFE3 gene fusion of alveolar soft part sarcoma. *The American Journal of Pathology*.

[6] Argani P., Antonescu C. R., Couturier J. (2002). PRCC-TFE3 renal carcinomas. *The American Journal of Surgical Pathology*.

[7] Clark J., Lu Y.-J., Sidhar S. K. (1997). Fusion of splicing factor genes PSF and NonO (p54^nrb^) to the TFE3 gene in papillary renal cell carcinoma. *Oncogene*.

[8] Calió A., Grignon D. J., Stohr B. A., Williamson S. R., Eble J. N., Cheng L. (2017). Renal cell carcinoma with TFE3 translocation and succinate dehydrogenase B mutation. *Modern Pathology*.

[9] Xia Q.-Y., Wang Z., Chen N. (2017). Xp11.2 translocation renal cell carcinoma with NONO-TFE3 gene fusion: morphology, prognosis, and potential pitfall in detecting TFE3 gene rearrangement. *Modern Pathology*.

[10] Pivovarcikova K., Grossmann P., Alaghehbandan R., Sperga M., Michal M., Hes O. (2017). TFE3-fusion variant analysis defines specific clinicopathologic associations amog Xp11 translocation cancers. *The American Journal of Surgical Pathology*.

[11] Argani P., Zhong M., Reuter V. E. (2016). TFE3-fusion variant analysis defines specific clinicopathologic associations among Xp11 translocation cancers. *The American Journal of Surgical Pathology*.

[12] Magers M. J., Udager A. M., Mehra R. (2015). MiT family translocation-associated renal cell carcinoma: a contemporary update with emphasis on morphologic, immunophenotypic, and molecular mimics. *Archives of Pathology & Laboratory Medicine*.

[13] Smith N. E., Illei P. B., Allaf M. (2014). t(6;11) renal cell carcinoma (RCC). *The American Journal of Surgical Pathology*.

[14] Argani P., Lui M. Y., Couturier J., Bouvier R., Fournet J.-C., Ladanyi M. (2003). A novel CLTC-TFE3 gene fusion in pediatric renal adenocarcinoma with t(X;17)(p11.2;q23). *Oncogene*.

[15] Huang W., Goldfischer M., Babayeva S. (2015). Identification of a novel PARP14-TFE3 gene fusion from 10-year-old FFPE tissue by RNA-seq. *Genes, Chromosomes and Cancer*.

[16] Malouf G. G., Su X., Yao H. (2014). Next-generation sequencing of translocation renal cell carcinoma reveals novel RNA splicing partners and frequent mutations of chromatin-remodeling genes. *Clinical Cancer Research*.

[17] Classe M., Malouf G. G., Su X. (2017). Incidence, clinicopathological features and fusion transcript landscape of translocation renal cell carcinomas. *Histopathology*.

[18] Wang X.-T., Xia Q.-Y., Ye S.-B. (2018). RNA sequencing of Xp11 translocation-associated cancers reveals novel gene fusions and distinctive clinicopathologic correlations. *Modern Pathology*.

[19] Antic T., Taxy J. B., Alikhan M., Segal J. (2017). Melanotic translocation renal cell carcinoma with a novel ARID1B-TFE3 gene fusion. *The American Journal of Surgical Pathology*.

[20] Argani P., Lal P., Hutchinson B., Lui M. Y., Reuter V. E., Ladanyi M. (2003). Aberrant nuclear immunoreactivity for TFE3 in neoplasms with TFE3 gene fusions. *The American Journal of Surgical Pathology*.

[21] Lopez-Beltran A., Scarpelli M., Montironi R., Kirkali Z. (2006). 2004 WHO classification of the renal tumors of the adults. *European Urology*.

[22] Argani P., Aulmann S., Karanjawala Z., Fraser R. B., Ladanyi M., Rodriguez M. M. (2009). Melanotic Xp11 translocation renal cancers. *The American Journal of Surgical Pathology*.

[23] Pei J., Cooper H., Flieder D. B. (2019). NEAT1-TFE3 and KAT6A-TFE3 renal cell carcinomas, new members of MiT family translocation renal cell carcinoma. *Modern Pathology*.

[24] Motyckova G., Weilbaecher K. N., Horstmann M., Rieman D. J., Fisher D. Z., Fisher D. E. (2001). Linking osteopetrosis and pycnodysostosis: regulation of cathepsin K expression by the microphthalmia transcription factor family. *Proceedings of the National Academy of Sciences*.

[25] Martignoni G., Pea M., Gobbo S. (2009). Cathepsin-K immunoreactivity distinguishes MiTF/TFE family renal translocation carcinomas from other renal carcinomas. *Modern Pathology*.

[26] Martignoni G., Gobbo S., Camparo P. (2011). Differential expression of cathepsin K in neoplasms harboring TFE3 gene fusions. *Modern Pathology*.

[27] Rao Q., Williamson S. R., Zhang S. (2013). TFE3 break-apart FISH has a higher sensitivity for Xp11.2 translocation-associated renal cell carcinoma compared with TFE3 or cathepsin K immunohistochemical staining alone. *The American Journal of Surgical Pathology*.

[28] Malik S., Roeder R. G. (2010). The metazoan mediator co-activator complex as an integrative hub for transcriptional regulation. *Nature Reviews Genetics*.

[29] Boube M., Joulia L., Cribbs D. L., Bourbon H.-M. (2002). Evidence for a mediator of RNA polymerase II transcriptional regulation conserved from yeast to man. *Cell*.

[30] Shaikhibrahim Z., Offermann A., Halbach R. (2015). Clinical and molecular implications of MED15 in head and neck squamous cell carcinoma. *The American Journal of Pathology*.

[31] Shaikhibrahim Z., Menon R., Braun M. (2014). MED15, encoding a subunit of the mediator complex, is overexpressed at high frequency in castration-resistant prostate cancer. *International Journal of Cancer*.

[32] Offermann A., Vlasic I., Syring I. (2017). MED15 overexpression in prostate cancer arises during androgen deprivation therapy via PI3K/mTOR signaling. *Oncotarget*.

[33] Weiten R., Müller T., Schmidt D. (2018). The mediator complex subunit MED15, a promoter of tumour progression and metastatic spread in renal cell carcinoma. *Cancer Biomarkers*.

[34] Ellis C. L., Eble J. N., Subhawong A. P. (2014). Clinical heterogeneity of Xp11 translocation renal cell carcinoma: impact of fusion subtype, age, and stage. *Modern Pathology*.

[35] Pflueger D., Sboner A., Storz M. (2013). Identification of molecular tumor markers in renal cell carcinomas with TFE3 protein expression by RNA sequencing. *Neoplasia*.

[36] Caliò A., Segala D., Munari E., Brunelli M., Martignoni G. (2019). MiT family translocation renal cell carcinoma: from the early descriptions to the current knowledge. *Cancers*.

[37] Camparo P., Vasiliu V., Molinie V. (2008). Renal translocation carcinomas. *The American Journal of Surgical Pathology*.

[38] Damayanti N. P., Budka J. A., Khella H. W. Z. (2018). Therapeutic targeting of TFE3/IRS-1/PI3K/mTOR axis in translocation renal cell carcinoma. *Clinical Cancer Research*.

